# “We Want Our Therapist to Talk With Us About Sexuality and Gender Diversity”: Qualitative Perspectives of Adolescents and Professionals in Youth Mental Health Care

**DOI:** 10.1177/13591045261421742

**Published:** 2026-02-07

**Authors:** Sara Bungener, Anouk Verveen, Thomas Steensma, Annelou de Vries, Arne Popma, Bernadette Hennipman, Anja de Kruif

**Affiliations:** 1 Center of Expertise on Gender Dysphoria, Amsterdam University Medical Centers, location VUmc, Amsterdam, the Netherlands; 2 Levvel, Academic Center for Child and Adolescent Psychiatry, Amsterdam, the Netherlands; 3 Department of Child and Adolescent Psychiatry, Department of Medical Psychology, Amsterdam University Medical Centers, location VUmc, Amsterdam, the Netherlands; 4 Department of Methodology and Applied Biostatistics, Faculty of Earth and Life Sciences, VU University Amsterdam, Amsterdam, The Netherlands

**Keywords:** adolescent mental health, sexuality, sexual orientation and gender identity (SOGI), LGBTQ+ youth, psychotropic medication, sexual side effects, clinical communication: sexual abuse

## Abstract

**Aims:**

Youth receiving mental health care often face challenges related to romantic relationships, sexuality, and sexual orientation and gender identity (SOGI). Although widely recognized as relevant, these topics are infrequently addressed in youth mental health care. This study explored how youth and mental health professionals experience discussions about sexuality and SOGI in clinical practice.

**Methods:**

In-depth interviews were conducted with 21 youth aged 15–22 years receiving psychiatric care, alongside three focus groups with 20 mental health professionals, at a youth mental health center. Data were analyzed using thematic analysis.

**Results:**

Youth described romance, sexuality, and identity as a normal part of life and closely linked to their well-being, while mental health difficulties often complicated relationships and identity processes. Professionals reported hesitation in raising these topics due to practical and personal barriers. Cultural and religious contexts shaped experiences, particularly for LGBTQ+ youth. Both groups emphasized the importance of addressing sexual side effects of psychotropic medication.

**Conclusion:**

This study highlights a gap between the recognized relevance of sexuality and SOGI and their discussion in everyday practice. Youth want these topics addressed in mental health care conversations. Bridging this gap requires proactive, youth-centered and culturally sensitive communication, supported by training and institutional attention.

## Plain Language Summary

Youth is a stage when young people explore relationships, sexuality, and questions about sexual orientation and gender identity (SOGI). These experiences are central to well-being, but for youth with mental health conditions they can be more difficult. Symptoms and medications may affect intimacy, and LGBTQ+ youth may face stigma or rejection, especially in cultural or religious settings. Despite their importance, these topics are rarely addressed in youth mental health care. This study explored the perspectives of adolescents and mental health professionals (MHPs). We interviewed 21 youth (15–22 years) in psychiatric care and held three focus groups with 20 MHPs (26–55 years) at a youth mental health center in Amsterdam. We analyzed their views on sexuality, gender, and communication in treatment. Youth said romantic and sexual experiences could boost confidence but also cause insecurity or harm, especially without consent. Mental health problems often made it harder to form healthy relationships. LGBTQ+ youth described added stress from stigma and lack of acceptance. Several reported sexual side effects from medication that were not discussed with them. Cultural and religious influences could be either challenging or protective. Conversations about sexuality and gender identity were strongly desired by youth. Both groups agreed that professionals, not patients, should initiate them, in private and with clear explanations. Barriers included clinician discomfort, parents in the room, and uncertainty about language. Facilitators included trust, training, and supportive environments. Respectful, confidential conversations about sexuality, gender, and medication side effects should be a routine part of youth mental health care. Professional-initiated dialogue can build trust, improve engagement, and better support.

## Introduction

Adolescence and young adulthood are developmental stages marked by rapid physical, emotional, social, and cognitive changes (Sawyer et al., 2018; World Health Organization, 2022). During this period, most young people begin to explore their sexuality: falling in love, forming romantic relationships, experiencing intimacy, and reaching key sexual milestones, including their first solo or partnered sexual experiences (Baams et al., 2015; de Graaf et al., 2018; Kaestle et al., 2021; Kann et al., 2016; Lefkowitz et al., 2018; Lindberg et al., 2021; Mercer et al., 2013; Rotermann & McKay, 2020; Scharmanski & Heßling, 2021; Shulman et al., 2017; Tolman & McClelland, 2009; World Health Organization, 2025). As part of this broader development, many also begin to form core aspects of their identity, including sexual orientation and gender identity (SOGI), which are closely linked to emotional well-being and self-understanding (Mousavi et al., 2025). Following the World Health Organization, “adolescents” are typically defined as those aged 10–19 years and “young adults” as those aged 20–24 years. In this paper, we use the term “youth” to refer specifically to the young people aged 15–22 who participated in our study and who receive specialized youth mental health care. When we use the terms “adolescents” or “young adults” in the Introduction and Discussion, we follow the age ranges used in the cited literature.

All adolescents and young people should have the opportunity to explore and develop their sexuality and identity in a healthy and affirming way (World Health Organization, 2025). However, adolescents with mental health disorders often face additional barriers. Around 15% of adolescents globally are diagnosed with a mental health condition during this critical phase of development (Agency for Healthcare Research and Quality (US), 2022). Despite the relevance of sexuality to youth mental health, research and clinical practice often focus on risks and dysfunction, overlooking its positive and developmental aspects (Harden, 2014; Kågesten & van Reeuwijk, 2021). In the context of mental health care, some concerns arise: Conditions such as anxiety, depression, trauma-related disorders, psychosis, and neurodevelopmental conditions may impair emotional regulation, communication, desire, and interpersonal functioning, all essential to healthy sexual development (Choukas-Bradley et al., 2020; Dewinter et al., 2017; Hebert et al., 2013; Hortal-Mas et al., 2020; Longmore et al., 2004; Marsh et al., 2015; Montesi et al., 2013; Torralbas-Ortega et al., 2023). Psychotropic medications can cause sexual side effects, such as reduced desire and anorgasmia, which are frequently left unaddressed (Levine & McGlinchey, 2015; McMillan et al., 2017; Scharko, 2004). Youth in mental health care are at higher risk of having experienced sexual abuse in the past or present, estimated between 10% and 40%, with many cases remaining undisclosed for years due to fear, shame, or lack of support (Barth et al., 2013; Knipschild et al., 2024; Rossiter et al., 2015; Skar et al., 2019). LGBTQ+ (lesbian, gay, bisexual, transgender, queer or questioning, used as an umbrella term for sexual and/or gender identities outside heterosexual and cisgender norms, and “+” referring to other sexual and gender minority identities not explicitly listed) topics are particularly important in youth mental health care, as many young people identify as LGBTQ+ (around 9.5–18%) or are in the process of questioning their sexual orientation or gender identity (Alaggia et al., 2019; Conron, 2020; Marino et al., 2024; Phillips et al., 2024). Compared to their cisgender and/or heterosexual peers, they are more likely to experience discrimination and exclusion in their environments, which contributes to an increased risk of mental health challenges, including suicidal thoughts and behaviors, and these difficulties may be intensified in cultural or religious settings where LGBTQ + identities are not accepted (Collier et al., 2013; de Lange et al., 2021; Mondave et al., 2025; Rossman et al., 2017).

Despite the relevance of sexuality and SOGI to youth development and mental health, these topics are often overlooked in psychiatric care (de Lange et al., 2021; McMillan et al., 2017; Sonneveld & Bungener, 2022). In our previous study, nearly all youth mental health professionals expressed the importance of these topics (99.5%), yet few discussed sexuality (19.9%) or gender identity (2.8%) regularly (Bungener et al., 2022). Sexual side effects of medication were addressed with only 20.3% of patients. Common barriers are limited knowledge, provider discomfort, and assumptions about youth embarrassment. These patterns mirror international findings across youth general health care and adult mental health care settings (Alexander et al., 2014; Boekeloo, 2014; Bungener et al., 2022; Rele & Wylie, 2007; Voermans et al., 2012).

Most research focuses on provider views or general health settings, instead of the perspectives of youth in psychiatric care, leaving a gap in understanding how young people actually experience, or want to experience, conversations about sexuality and gender in mental health contexts (Alexander et al., 2014; de Lange et al., 2021).

This qualitative study addresses these gaps by examining both youth and professional perspectives on communication about romance, sexuality, and gender identity in youth mental health care. We aim to understand how these topics are experienced and navigated in the context of mental health, whether young people wish to discuss them during treatment, and, if so, how communication can be improved to better support both youth and professionals in these conversations.

## Methods

### Study Design and Setting

This qualitative study was part of the SexQ project and was conducted at Levvel, a multidisciplinary academic institution in Amsterdam, the Netherlands, providing specialized mental health care for children, adolescents and youth aged 0 to 23 years and their families.

### Participants and Recruitment

Participants included two groups: (1) youth aged 15 to 22 currently receiving psychiatric care and (2) mental health care professionals (MHPs) currently working with youth aged 12 to 22. Throughout this article, we use the term “youth” or “young people” to refer to the 15–22-year-old patients in our sample.

For the inclusion of youth participants, treating clinicians were asked via advertisements (intranet and email) to inform their patients about the project. They informed youth patients about the study using standardized information materials (digital flyer, information folder). Interested individuals were then contacted by telephone by a research assistant. To preserve confidentiality and respond to participant preferences, youth were offered a choice of face-to-face (*n* = 11) or video interviews (*n* = 10), and this preference was collected verbally during scheduling. Individual interviews were chosen over focus groups due to the sensitivity of the topics discussed. Written informed consent was obtained from all participants. For minors (under 16 years), both written youth and parental consent were required. Inclusion criteria for adolescents were: (1) current treatment at Levvel, (2) age 15–22, and (3) fluency in Dutch. The selected age range was intended to include both middle adolescents and youth who still fall under youth care services in the Dutch mental health system. Recruitment followed a purposive sampling strategy to ensure heterogeneity in gender identity, sexual orientation, psychiatric diagnosis, and cultural or religious background. Of the 24 young people invited, 21 participated. Three did not participate due to clinical instability (1) or non-response (2) (see Table 1 for demographics).

**Table 1.** General Characteristics of Youth Recieving Mental Health Care

Variabele	All participants
	*N* = 21 (%)
Age at assesment	
*M (SD)*	17.6 (1.9)
Range (years)	15.9–22.00
Gender	
Female	9 (42.9)
Male	5 (23.8)
Transgender	2 (9.5)
Non-binary	2 (9.5)
Questioning	3 (14.3)
Sexual orientation	
Heterosexual	8 (38.1)
Lesbian/homosexual	3 (14.3)
Bisexual/bicurious	5 (23.8)
Pansexual	3 (14.3)
Questioning	2 (9.5)
Sexual experienced^a^	
Experieneced	11 (52.4)
Not experienced	10 (47.6)
Diagnosis of youth^b,c,d^	
ADHD	4 (19.0)
ASD	6 (28.6)
Anxiety/OCD	5 (23.8)
Trauma	3 (14.3)
Mood disorder	12 (57.1)
Eating disorder	3 (14.3)
Somatic symptomen disorder	1 (4.8)
Birthplace of parents^e^	
Netherlands	12 (57.1)
Europe	2 (9.5)
Asia	3 (14.3)
Africa	3 (14.3)
South America	1 (4.8)
Religion	
None	15 (71.4)
Islam	2 (9.5)
Christian	2 (9.5)
Jewish	1 (4.8)
Hindu	1 (4.8)

*Note:* ADHD = Attention Deficit Hyperactivity Disorder; ASD = Autism Spectrum Disorder; OCD = Obsessive Compulsive Disorder.

^a^Sexual experienced was defined as “all types of sexual activity as experienced by the participant” excluding (child) sexual abuse.

^b^Multiple answer options.

^c^Diagnosis as described by youth.

^d^Diagnosis according to the DSM-5 (Diagnostic and Statistical Manual of Mental Disorders).

^e^Birthplace of (one of the) birthparents, by continent: Europe (Greece, Italy); Africa (Egypt, Marocco); Asia ( Indonesia, China, Israel); South America (Surinam).

Mental health professionals (MHPs) were recruited through internal announcements and team meetings. Eligible participants were actively practicing clinicians working with youth aged 12–22 and fluent in Dutch. To ensure a heterogeneous sample, we included professionals from a broad age range and diverse cultural backgrounds, representing various disciplines including psychology, psychiatry, nursing, social work, and systemic therapy. Three focus groups were conducted, involving a total of 20 MHPs. Written informed consent was obtained from all participants (see Table 2 for demographics).

**Table 2.** General Characteristics of Mental Health Care Professional (MHP)

Variabele	All participants
	*N* = 20 (%)
Age at assessment	
*M ( SD)*	36.2 (7.9)
Range (years)	26–55
Gender, *N* (%)	
Female	17 (85.0)
Male	3 (15.0)
Profession	
Psychologist (healthcare, basis, clinical)	8 (40)
Psychotherapist	2 (10)
Psychiatrist	2 (10)
Resident (MD)^a^	3 (15)
System therapist	3 (15)
Social worker	2 (10)

*Note*: ^a^Medical doctor specializing in psychiatry.

### Study Procedure

The individual interviews with youth participants and the focus groups with MHPs were held from April 2020 to November 2021. Interviews lasted 30–70 minutes and were conducted in private with one interviewer. Youth received a €20 voucher. MHP focus groups lasted 75–90 minutes and were conducted using a dual-moderator format, with two facilitators guiding the discussion. All digital recordings were anonymized and deleted after transcription.

We used a reciprocal triangulation procedure: Themes from youth interviews were presented during MHP focus groups, and MHP responses were later shared with adolescents for reflection. This reciprocal procedure aimed to triangulate data and validate interpretations (Patton, 1999). Participation had no effect on care. The study was approved by the Medical Ethics Committee of the Amsterdam UMC, location VUmc. All interviews and focus groups were conducted and transcribed in Dutch. Coding and theme development were based on the original Dutch transcripts. For publication, illustrative quotations were translated into English by a researcher and checked by a native English-speaking editor, aiming to preserve meaning and tone rather than literal wording.

### Interview Description

The multidisciplinary research team included three psychiatrists, one certified sexologist, one qualitative methods professor, two gender diversity researchers and three trained research assistants. Roles were based on formal training and professional expertise. Two distinct semi-structured interview guides were developed for adolescents and MHPs informed by prior literature and expert input (S. B., A. K. and B. v. B.), which we iteratively updated to include topics that emerged during the interviews and focus groups (e.g., more questions addressing cultural and religious influences were added based on participant feedback) (Bungener et al., 2022; Choukas-Bradley et al., 2020; Dewinter et al., 2017; Harden, 2014; Hebert et al., 2013; Kaestle et al., 2021; Longmore et al., 2004; Marsh et al., 2015; McMillan et al., 2017; Montesi et al., 2013; Torralbas-Ortega et al., 2023). A pilot test with two youth participants assessed clarity and relevance of questions. Youth were asked about experiences with falling in love, relationships, sexuality (including sexual orientation) and gender identity in the light of mental health, experiences, perceived relevance and needs in therapeutic conversations on sexuality and gender. MHPs were asked about communication practices, perceived competencies, and professional challenges. Both guides included probes on medication side effects, culture, and religion (see Table 3 for sample interview prompts of youth and MHPs).

**Table 3.** Sample Questions From Interview Guides on Youth and MHPs

Topics queried	Example questions youth and MHP
Youth experiences with romance and sexuality	• How would you describe the role of falling in love and romantic relationships in your life?
	• How would you describe the role of sexuality in your life?
	• Have you ever questioned your sexual orientation or gender identity? What was that process like for you? What was the influence on your mental health?
	• How have your cultural or religious background and values influenced how you think or feel about sexuality or gender identity?
Youth experiences in communication	• Has anyone in mental health care ever talked with you about things like sex, relationships, or identity? What was that like for you?
	• How would you feel if your therapist or doctor brought up side effects of medication related to sex or relationships?
MHPs experiences in communication	• Tell us about your experiences in discussing sexuality and romance with youth.
	• What were barriers and facilitators that you faced in talking about these topics?
Recommendations for future health care	• Should practitioners discuss the topic of sexuality? (can you explain your answer)
	• If you could give advice to mental health professionals about talking to youth about sexuality and identity, what would you say?
	• Is there anything else you think mental health professionals should know about what young people need when it comes to talking about sexuality and identity?

*Note:* MHP = Mental Health Care Professional.

### Data Analysis

All interview and focus group data were transcribed verbatim. Data were analyzed using the principles of thematic analysis by Braun and Clarke and MAXQDA (version 2022.2.1) analysis software (Braun & Clarke, 2006, 2021 (online 2020)). In line with their later work, we adopted a team-based, codebook-oriented form of thematic analysis, which was appropriate for the applied clinical context and the multidisciplinary research team. Analysis followed the six phases described by Braun and Clarke: familiarization, initial coding, construction and review of candidate themes, defining and naming themes, and reporting. Familiarization involved repeated reading of transcripts and team discussions. Coding was primarily inductive and semantic, informed by the study aims. The first 11 transcripts were reviewed individually by two members (A. V. and S. B.) and the last 10 by three members of the research team (A. V., S. B. and B. v. B.). The members independently generated the preliminary codes. A shared codebook was developed and refined through analytic discussions. Rather than aiming to establish objectivity, these discussions served to support transparency, shared understanding and critical reflection, consistent with quality criteria for team-based thematic analysis. (Braun & Clarke, 2021). Themes were developed iteratively by moving between codes, themes, and the full dataset. Data collection and analysis proceeded concurrently and continued until no substantially new insights relevant to the research questions emerged and sufficient conceptual depth was achieved.

## Results

A total of three main themes and eleven subthemes were identified from the 21 in-depth interviews with youth and the three focus groups with 20 MHPs. These three main themes were: (I) the interplay between mental health and sexuality, sexual orientation, and gender identity; (II) experiences with and needs regarding communication on sexuality and gender identity during treatment; and (III) recommendations to enable communication (see Table 4). We use the term “peers” to refer to other young people of a similar age in the general population (e.g., classmates, friends) of the participants, regardless of whether they receive mental health care.

**Table 4.** Youth Recommendations to Enable Communication Regarding Sexuality and Gender Identity

Make sure parents are out of the room.
Secure privacy and confidentiality.
Ask permission and explain the reason for asking.
Never force someone to speak.
Initiate the conversation, don’t wait for youth to start.
Use inclusive language.
Be aware of the patient’s cultural and faith background.
Recognize that “coming out” is not a wanted (or safe) option for everyone.
Support identity exploration without forcing the issue.

### Interplay Between Mental Health and Sexuality, Sexual Orientation, and Gender Identity

#### Normal Part of Life

Both youth and professionals emphasized that romantic relationships, sexual experiences (whether solo or with a partner), and intimacy play a large role in the everyday lives of youth “normal life,” just like their peers.

I also notice that at my age, that sexuality is quite a thing. Uncertainties and things like that… yes, it is just certainly a big thing during this age for a lot of young people. **Boy, 16** **years (heterosexual)**

#### Sexuality and Relationships’ Influence on Mental Health

Youth described how sexual activity could bring feelings of validation, belonging, or self-worth, but could also cause feelings such as insecurity, guilt, or emotional harm, particularly when experiences were non-consensual or crossed personal boundaries. These accounts highlight the complex and often ambivalent link between sexual experiences, relationships, and adolescents’ mental health.

Being sexually active gives some kind of validation that you are important or something. And that can really affect how you feel. In the short term but also in the long term. **Girl, 17** **years (heterosexual)**

What is interesting is that sex or masturbation really gives hormones that make you happy and stuff. **Girl, 17** **years (heterosexual)**

If you go beyond your own boundaries, then it is just a very bad feeling because you are not doing it for yourself and it can also feel as if you are being used. **Girl, 17 years (Asian background, heterosexual)**

#### Mental Health’s Influence on Sexuality and Relationships

Conversely, youth and MHPs reported that symptoms of mental illness, including depression, anxiety, trauma, and neurodevelopmental disorders, often made it harder to form or maintain healthy romantic relationships. Young people shared that insecurity or low self-esteem, often associated with their mental health condition, made them vulnerable to unhealthy or unsafe sexual relationships. Professionals confirmed seeing this pattern regularly in clinical practice.

I’ve also seen a lot of autistic guys lately who struggle with sexuality because they just think “No girl sees me because they think I'm autistic.” **Resident, male, 29 years**

I can be very insecure, especially when I am really, super depressed, then it really feels like nobody will like me or ever want me. **Boy, 16 years (heterosexual)**

First of all, that [sexual] trauma sucks, because it's not supposed to happen, but it can also make you afraid of ever having another sexual experience. **Girl, 16 years (heterosexual)**

We also regularly see young people who have experienced sexual trauma, I see this has a major influence on how you are in a relationship or in sexual behavior. **Psychologist, female, 32 years**

#### Sexual Orientation and Gender Identity Influence on Mental Health

Youth who identified as or questioned being LGBTQ + frequently expressed emotional distress related to societal and peer expectations, perceived stigma, and lack of acceptance in their social environment. Feelings of alienation or confusion about one’s sexual or gender identity were reported as contributing to anxiety or depressive symptoms.

I think some people are struggling with themselves because I am different or I am not what I am supposed to be. I think that itself can be very unpleasant... I speak from experience**. Girl, 16 years (bisexual)**

Well, the depression did not arise from it, but it was very much amplified by it. … Because I also no longer knew whether I wanted to be a woman or a man or something in between or nothing at all. And that made me very depressed because I simply no longer knew who or what I should be. **Transgender youth, 18 years (gay)**

I feel there are a lot of stigmas around gender identity, and that makes it difficult to cope. **Transgender youth, 17 years (African background, bisexual)**

MHPs echoed these concerns, noting that struggles with identity and mental health frequently intersect in LGBTQ + youth.

#### Sexual Side Effects of Psychotropic Medication

Several young people reported experiencing sexual side effects (e.g., reduced desire, arousal issues) related to psychotropic medication. Some said these side effects were never discussed by prescribing clinicians, while they and others stressed the value of open and transparent communication. One case involved a couple in which both partners were on medication, making it unclear whose treatment was causing the effects.

And suddenly my boyfriend’s libido was lower and he had trouble coming, which actually turned our entire sex life upside down. First, we didn’t know why, it was by medication. And if that drug has any sexual side effects, it must be included. Because it can really have a lot of impact on your mental health if something down there isn’t right. **Girl, 20 years (heterosexual)**

#### Cultural and Faith-Based Influences

Youth from diverse cultural and religious backgrounds described a wide range of additional challenges when navigating dating, sexuality, and questions on sexual orientation or gender identity. Experiences differed greatly across individuals. They included family expectations, cultural or religious rules, fear of social rejection, secrecy, risk of violence or exclusion, and longstanding taboos.

For some, religious beliefs created inner conflict and added to mental health struggles. For others, culture and faith provided strength. The provided examples illustrate how supportive families and faith could give guidance, or a sense of belonging during a difficult stage of development and identity searching.

Mental health professionals noted that gaps in their own cultural awareness sometimes limited effective care and made it harder to build open communication with youth.

I think it is good for professionals to discuss this with them because it is quite a taboo with many people. [..] At least in my culture, I never talk about it either. My parents and family have never talked about LGBT literally. **Girl, 17 years (Asian background, heterosexual)**

My family can never ever know I am a lesbian, they would never tolerate me**. Girl, 22 years (South American background, bicurious)**

I think if you come from one culture, it's harder to get to understand other cultures and specific parts. **Social worker, female, 38 years**

### Communication Experiences and Needs

#### Frequency and Quality of Conversations

Most young people said that sexuality and gender identity were rarely, if ever, brought up in treatment. When these topics did come up, youth and MHPs noted they were usually limited to situations tied to psychiatric problems, such as sexual trauma. Youth often described the conversations as brief, awkward, or superficial. A smaller number recalled positive experiences, including when the MHP initiated the discussion, used inclusive language, or created a sense of trust. MHPs acknowledged avoiding these conversations despite recognizing their relevance.

I did have the question whether I had lost my virginity and that [conversation] remained really very short. It's just more like “Have you ever lost your virginity?” Yes. “How was that?” Good. And that's it. **Girl, 17 years (heterosexual)**

Very strange, but I think that in psychiatry, there is quite a big taboo on sexuality, while that is not what you would expect. **Psychologist, female, 28 years**

#### Who Needs the Conversations

Youth who were questioning their gender identity, experiencing sexual distress, or navigating romantic and sexual development expressed a strong need for open and respectful dialogue with their MHP.

The symptoms of depression I experienced were at the base linked to gender identity, so I would have preferred to discuss that more. **Transgender youth, 18 years (gay)**

For me, starting dating and first things like kissing gave me huge insecurities and a lot of negative thoughts, while in therapy the only social circles we discussed were my parents or friends. [..] It would have helped to talk about these. **Girl, 17 years (heterosexual)**

They emphasized that such conversations can bring clarity, validation, and emotional support. MHPs generally agreed that these issues should be treated as a normal part of adolescent care.

I think they also go on with unhealthy relations for a very long time without anything being done about it, especially during puberty and young adulthood. So I think we really need to do something about that. **Psychologist, male, 34 years**

#### Who May Not Need It—Yet

Some young people without current sexual experience felt that in-depth discussion was not necessary at the moment for them. Still, they recommended that MHPs raise the topic to normalize it and ensure support is available for peers who might need it. They also valued knowing the subject could be revisited later during the treatment, when more relevant.

I don’t have any questions on sexuality now, but maybe in the next years I will, and then I know we can talk about it. **Boy, 17 years (heterosexual)**

Well, it wasn't necessary for me, but I think if you don't ask at all, that you miss it with the people who do need it. So I think it is an important question. **Girl, 16 years (questioning sexual orientation)**

#### Medication Side Effects: to Talk or Not?

All youth supported proactively discussing sexual side effects when prescribing psychotropic medication. They stressed that awareness of potential effects would not erode trust—if anything, it could strengthen it. Some MHPs, however, hesitated to bring up these concerns, fearing that youth might refuse medication.

People can lose confidence in the practitioner or in medicines if not told. That is also about trust. **Girl, 16 years (bisexual)**

You can also not talk about it because you are afraid. That they don't take the pills at all if they also know that it can cause sexual complaints. **Psychiatrist, male, 45 years**

#### Barriers and Facilitators for Communication

MHPs pointed to several barriers: parents being present in sessions, limited time, personal discomfort, assumptions about adolescent discomfort, fear of saying the wrong thing, uncertainty about appropriate language, and a tendency to only address sexuality when directly tied to the presenting issue.

Whether you have had that in your education, but also whether you have learned to talk about these subjects in your own upbringing. **System therapist, female, 37 years**

If there is no direct link to sexuality, such as known trauma, then I tend to forget a little to ask it. **Psychologist, female, 34 years**

For me, I also think out of fear because you don't want to make someone feel weird...or because you don't want to expose someone's vulnerabilities by force. Yes, how do you actually do that? **Psychotherapist, female, 37 years**

I think it also plays a part in my expectation that it is a complicated subject for them. **Psychiatrist, female, 39 years**

I also think the age difference can make it more uncomfortable. Yes, what do I still know about the standards nowadays, am I such an old guy who is going to say a bit how it should be. You don't want to be a moral knight. **Psychologist, male, 52 years**

Facilitators included a strong therapeutic relationship, training on sexuality and gender diversity, a supportive clinic environment, and moments when adolescents themselves initiated the discussion.

It helps if a young person starts talking about it themselves. I mean, some young people are just throwing everything in themselves. **Psychologist, female, 34 years**

Youth, however, challenged some of these barriers. They noted that time is generally available in mental health care, and that discomfort often stems from the MHP’s own unease. They strongly supported excluding parents from these conversations and emphasized the importance of trust. At the same time, they disagreed with the idea that responsibility for starting the conversation should be on them.

### Recommendations to Enable Conversations

Youth and MHPs agreed that professionals, not patients, should initiate discussions on sexuality and gender identity.

I think the practitioner should bring that up, because I know it is very difficult to bring up problems at all for us. **Non-binary youth, 16 years (pansexual)**

It’s especially important that you make them feel like there is an opening to talk about it if they want to. That they know it is not taboo for us. **Psychologist, female, 27 years**

Youth preferred to have these discussions privately, without parents or others present; MHPs agreed. They also stressed the importance of explaining the rationale for talking about these topics, to avoid discomfort or misunderstanding. Creating a safe, confidential environment where youth feel respected and not pressured was emphasized.

Because if you don’t explain it, people will think, “Why do you want to know if I had sex?” **Girl, 17 years (bisexual)**

While opinions varied on when to start such conversations (whether at intake or after building trust during a treatment period) participants agreed that timing should be guided by the patient’s comfort and developmental (age) stage. Youth also noted that the MHP’s characteristics (e.g., gender, openness, lived experience) could make a difference in the comfort level of the conversation.

Well, I think things like how you want to be addressed [as gender], that that is useful to do directly at the intake. But I think about real sexuality, it is useful to wait until you get to know each other a little better. **Girl, 17 years (heterosexual)**

It is really easier to talk with a woman about female body symptoms than with men.’ **Girl, 16 years (heterosexual)**

If I could find a therapist who was non-binary myself, I would love it so much. Absolutely! Maybe you will then dare to open up more because you feel more equal. **Non-binary youth, 16 years (pansexual)**

Youth from diverse backgrounds advised MHPs to adopt a culturally and faith-sensitive approach, one that is supportive rather than judgmental. They recommended that professionals take the time to understand the cultural and religious context of the young person, including the social (family) expectations, norms, and positive cultural or faith factors that could influence their experiences.

## Discussion

This study explored young people receiving mental health care and mental health professionals (MHPs) experiences and perspectives regarding communication about romance, sexuality and gender identity during treatment. While both groups recognized these topics as relevant and appropriate within mental health care, their narratives revealed a persistent gap between acknowledged importance and everyday clinical practice. Youth framed sexuality and identity as normal, meaningful, and closely connected with their mental well-being, whereas MHPs described these topics through narratives of caution, uncertainty, discomfort and practical challenges. Together, these perspectives help to explain why conversations about sexuality and gender identity remain limited in practice despite their perceived importance. Youth shared the ways in which their mental well-being influenced their romantic relationships and sexual experiences and vice versa. They also offered insight regarding the mental impacts of struggling with sexual orientation and gender identity. While MHPs also expressed a desire to communicate about these topics with patients, a variety of underlying facilitating and inhibiting factors for communication were reported by both groups. In addition, relevant points related to cultural and religious background of youth emerged from the interviews.

While previous studies reported low frequencies of such conversations (2%–20% in youth psychiatry, 24%–43% in adult psychiatry) this study adds new depth by revealing the lived experiences behind those numbers (de Lange et al., 2021; Sonneveld & Bungener, 2022; Voermans et al., 2012). By focusing on participants’ accounts, our findings help explain why these conversations remain infrequent despite their perceived importance. Youth participants in our study positioned romance and sexuality (and for some identity) as normal and meaningful aspects of their everyday lives, echoing romance and sex experiences in youth general population studies (Baams et al., 2015; de Graaf et al., 2018; Kaestle et al., 2021; Kann et al., 2016; Lefkowitz et al., 2018; Lindberg et al., 2021; Mercer et al., 2013; Rotermann & McKay, 2020; Sawyer et al., 2018; Scharmanski & Heßling, 2021; Shulman et al., 2017; Tolman & McClelland, 2009; World Health Organization, 2022, 2025). Yet they reported that these topics are rarely addressed during mental health care, even though many view therapy as the ideal space for such discussions, in line with professional opinions. (Stevenson, 2004).

Youth described a wide range of both positive and negative influences of romantic relationships and sexuality on their mental well-being. Their accounts shows that romance and sexuality can be both supportive and challenging. Positive influences included feelings of validation, release of pleasant hormones, and boosting of self-esteem These experiences align with recent longitudinal studies in general youth, which show a significant increase in body image and self-esteem and comparatively low rates of psychological distress among sexually active young people (Horne & Zimmer-Gembeck, 2006; Vasilenko et al., 2012; Zimmer-Gembeck et al., 2011). Youth sexual health has also been associated with general well-being in early and later adulthood (Långström & Hanson, 2006). At the same time, participants described potential negative effects of sexuality and relationships, such as the emotional impact of romantic or sexual rejection, relationship breakups, lowered self-esteem and insecurity during sexual activities. In general, problems related to relationships and sexuality can have a profound impact on youth well-being and quality of life (Rosen et al., 2004; Scharko, 2004; Williams et al., 2010). Conversely, youth shared the ways in which their mental health problems could influence their ability to find love or enjoy sex. These effects could be directly related to psychiatric disorders (e.g., anxiety, depression, trauma or autism spectrum disorder) or indirectly related, as factors associated with the disorder (e.g., low self-esteem, enduring mental health-related stigma, and social exclusion) may interfere with young people’s ability to form relationships and obtain sexual experiences (Choukas-Bradley et al., 2020; Dewinter et al., 2017; Hebert et al., 2013; Longmore et al., 2004; Marsh et al., 2015; Montesi et al., 2013; Torralbas-Ortega et al., 2023; Wright et al., 2007).

Not all youth may need discussions about relationships and sexuality, as these topics may not be relevant to them at a given time, consistent with results observed in general youth healthcare (Elkington et al., 2013). However, youth participants emphasized that professionals in youth mental health care should still initiate these conversations broadly and proactively, as failing to do so could overlook those who are silently struggling with these issues. Additionally, the young people noted that they might have questions on these topics in the future (Elkington et al., 2013).

Youth expressed that explanation of the sexual side effects of psychotropic medication is just as necessary as explanations of sleep or weight gain side effects. While some MHPs expressed concern that youth might stop using medication if they knew about the possible sexual side effects, whereas youth framed transparent communication as essential for trust and shared decision-making. (Bungener et al., 2022; de Lange et al., 2021; Levine & McGlinchey, 2015; McMillan et al., 2017). Young people’s ability to trust that their MHP is providing them with correct information is essential for them and in line with adult literature: studies in adults show that open discussion of side effects, especially sexual side effects, and involving patients in treatment decisions significantly improve medication adherence, while poor communication is linked to early discontinuation (Brown & Bussell, 2016; Bull et al., 2002; Hoopes et al., 2017; National Institute for Health and Care Excellence (NICE), 2009).

Mental health care should be inclusive of and sensitive to all identities. LGBTQ + youth described the potentially significant mental health impact of questioning one’s sexual orientation or gender identity and their related mental healthcare needs, which are aligned with research findings in youth and adult LGBTQ + populations (Hall, 2018; Lucassen et al., 2017). Some young LGBTQ + participants from various cultural and religious backgrounds described the additional layers of stress, reflecting on the struggles and mental health problems that arise from the combination of being an LGBTQ + person with a certain (multi)cultural identity or religion in which LGBTQ + identities are not widely accepted. There is a complex interplay of these issues related to double minority stress, taboo, and exclusion, and youth participants in the present study stressed the importance of awareness of these complexities in mental health care (Shangani et al., 2020).

Youth participants indicated that conversations with their MHP are the designated place to talk about these sensitive subjects (McConnell et al., 2018). To understand the gap between the acknowledged importance and low frequency of communication, we examined the MHPs narratives identify underlying factors regarding such communication. Various personal and non-personal factors were reported, including MHPs’ feelings of shame, the presence of parents in the room, and lack of time, echoing similar findings in general youth health care and adult psychiatry (Alexander et al., 2014; Bungener et al., 2022; Rele & Wylie, 2007). In response, youth participants provided several recommendations to enable these conversations in a safe and inclusive manner (see table 4). For example, all youth, including those who were cisgender and/or heterosexual, said they would be comfortable with their MHP asking about their gender identity or sexual orientation if the reason for asking was explained. Voices suggests that treating sexuality and gender identity as routine aspects of care, rather than exceptional topics, may help bridge this gap. Centering youth voices suggest that treating sexuality and gender identity as routine topics in youth mental health care, rather than exceptional topics, may help to bridge this gap. Institutions should focus on increasing attention, knowledge, and practical skills regarding these topics in youth psychiatry, including in training and daily practice (Levine & Scott, 2010; Miller & Byers, 2010; Stevenson, 2004).

## Strengths and Limitations

One of this study’s strengths is that it brings together the perspectives of both young people and mental health professionals (MHPs), giving a fuller picture of how sexuality and gender identity are (or aren’t) addressed in youth mental health care. The use of interviews and focus groups also added depth, producing detailed accounts that are directly useful for clinical practice. However, the study has limitations. All participants came from the same urban area and the same youth mental health service, which limits how broadly the findings can be applied. We used purposive sampling to reduce the risk of including only those with a particular interest in sexuality and gender issues, and the sample included a wide age range of youth and MHPs. While the overall sample was large enough to capture the main themes and we continued recruiting until sufficient conceptual depth was achieved, the group of LGBTQ + youth from culturally and religiously diverse backgrounds was relatively small. This means that those findings should be interpreted with caution. More targeted research focusing on these populations is needed. Even so, the study offers practical recommendations for working with LGBTQ + youth from multicultural or faith-based backgrounds. Overall, the findings give insight into the needs of young people and professionals, showing how mental health, sexuality, and gender identity intersect, and underlining the importance of MHPs, not youth, initiating these conversations.

## Future Research Directions

Future work should look at how routine conversations about sexuality and gender identity affect outcomes such as wellbeing, treatment progress, and (medication) adherence over time. There is also a need to explore how culture, migration, and religion shape these experiences. Co-designing interventions with LGBTQ + youth, youth of color, and faith communities could help make these discussions more effective and reduce stigma. On a practical level, research on systemic barriers, such as policies, workloads, and clinician attitudes, could guide how services put this into practice.

## Conclusion

This study shows that although both young people and mental health professionals recognize romance, sexuality, and gender identity as relevant topics in youth mental health care, these issues are rarely addressed in practice. Youth framed sexuality and identity as normal and closely linked to their mental well-being, while professionals described hesitation and practical barriers. Bridging this gap requires proactive, youth-centered, and culturally sensitive communication, supported by training and institutional attention

## Data Availability

The datasets generated during and/or analyzed during the current study are available from the corresponding author on reasonable request.*
